# Pivotal role of vitamin D in mitochondrial health, cardiac function, and human reproduction

**DOI:** 10.17179/excli2022-4935

**Published:** 2022-07-20

**Authors:** Alavala Matta Reddy, Mumtaz Iqbal, Hitesh Chopra, Shaheda Urmi, Sunil Junapudi, Shabana Bibi, Santosh Kumar Gupta, Viajaya Nirmala Pangi, Inderbir Singh, Mohamed M. Abdel-Daim

**Affiliations:** 1 Department of Zoology, School of Life and Health Sciences, Adikavi Nannaya University, Rajahmundry 533296, Andhra Pradesh, India; 2 College of Arts and Science, University of South Florida, Tampa, FL33620, USA; 3 Chitkara College of Pharmacy, Chitkara University, Punjab140401, India; 4 Department of Pediatrics, Morsani College of Medicine, University of South Florida, Tampa, FL33612, USA; 5 Department of Pharmaceutical Chemistry, Geethanjali College of Pharmacy, Cherryal, Keesara, Medchalmalkajgiri District, Telangana, 501301, India; 6 Department of Biosciences, Shifa Tameer-e-Millat University, Islamabad, Pakistan; 7 Yunnan Herbal Laboratory, College of Ecology and Environmental Sciences, Yunnan University, Kunming 650091, China; 8 National Institute of Plant Genome Research, New Delhi 110067, India; 9 School of Life and Health Sciences, Adikavi Nannaya University, Rajahamahendravaram, Andhra Pradesh, India; 10 Department of Pharmaceutical Sciences, Pharmacy Program, Batterjee Medical College, P.O. Box 6231 Jeddah 21442, Saudi Arabia; 11 Pharmacology Department, Faculty of Veterinary Medicine, Suez Canal University, Ismailia 41522, Egypt

**Keywords:** Vitamin D, mitochondrial dysfunction, oxidative stress, cell damage, inflammation, cardiac diseases

## Abstract

Vitamin D, a secosteroid hormone, appears to have significant beneficial effects on various physiological systems, including the musculoskeletal system. Vitamin D assists in the regulation of numerous critical biological functions and physiological processes in humans, including inflammation, oxidative stress, and mitochondrial respiration, and is also linked to cardiac diseases. It is also reported that vitamin D plays a central role in molecular and cellular mechanisms, which reduce oxidative stress, and tissue damage and regulate cellular health. On the other side, hypovitaminosis D reduces mitochondrial activity and increases oxidative stress and inflammation in the body. Hypervitaminosis D increases the prevalence and severity of cellular damage. It has also been reported that vitamin D is involved in many functions of the reproductive system in human and critically play an important role in the reproductive tissues of women and men. Its role is very well defined, starting from female menarche to menopause, pregnancy, and lactation, and finally in male fertility. Hence, the appropriate amount of vitamin D is necessary to maintain the normal function of cell organelles. Based on recent studies, it is understood that vitamin D is involved in the biological activities of mitochondria in cells, especially in cardiomyocytes. In this review, we emphasized the role of vitamin D in mitochondrial respiration, which could significantly influence heart health and human reproduction.

## Introduction

**Figure 1:**
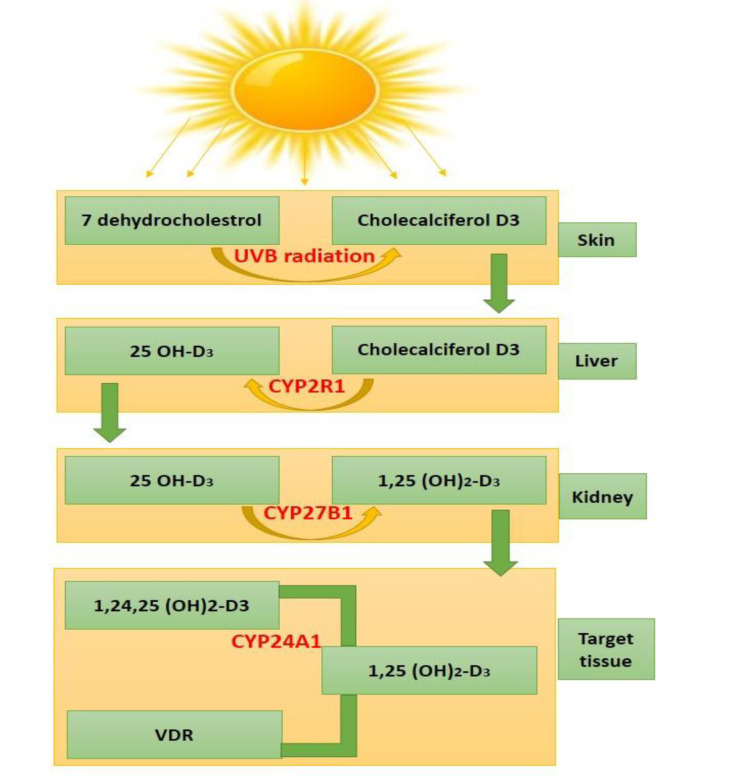
Vitamin D metabolism of human; an overview

Vitamin D is fat-soluble, linked to bone metabolism, and is well-known for its essential role in bones and skeletal muscle health (Halfon et al., 2015[55]). Vitamin D is present in bread, milk, fatty fish, mushrooms, and dietary supplements. As we know that there are two main forms of dietary or supplemental vitamin D. These are vitamin D2 and D3. Vitamin D2 is derived from plants, while vitamin D3 comes from animals (fatty fish or sheep's lanolin). Both the forms are significant for overall vitamin D levels. The biological roles of vitamin D are very well defined. Cholecalciferol (known as vitamin D3) is bound to serum vitamin D-binding protein (DBP). For the biological activation of vitamin D, two-step enzymatic pathways are necessary. These are involving 25-hydroxylase of the liver and 1α-hydroxylase (CYP27B1) of the kidney and extra-renal tissues. The majority of the functions are mediated by the VD receptor (VDR), which is a ligand-dependent transcription factor. This transcription factor is primarily localized in the nuclei of target cells. VDR works as a mediator for the genomic action of the biologically active hormone calcitriol (1,25(OH)_2_D3). Calcitriol is an active form of vitamin D, which is produced by the hydroxylation of 25(OH)D in kidneys under the regulation of parathyroid hormone (PTH) and serum calcium. This form acts as an inducer for the transcription of more than 900 genes (Minghetti and Norman, 1988[107]). VDR receptor is widely distributed over various tissues and organs including skeletal muscles, parathyroid glands, and the reproductive tissues. This indicates that various metabolic processes are regulated by VDR, significantly (Kinuta et al., 2000[78]) as shown in Figure 1(Fig. 1).

Vitamin D levels of 50 nmol/L were found to be insufficient in various clinical investigations and were linked to muscular atrophy and faintness (Tagliafico et al., 2010[152]; Van Langenberg et al., 2014[158]). When exposed to ultraviolet B rays, the human skin converts 7-dehydrocholesterol to vitamin D (Jäpelt and Jakobsen, 2013[66]; Wacker and Holick, 2013[160]). The *CYP P450* gene family has been expanded to include numerous genes that play pivotal roles in vitamin D activation and degradation (*CYP2R1, CYP27B1*, and *CYP24A1*). Vitamin D initially hydroxylated in the liver predominantly by the CYP2R1 enzyme to produce 25-hydroxyvitamin D and then in the kidneys by another enzyme, CYP27B1, to produce 1α,25-dihydroxyvitamin D, which is the hormonally active form (Griffin et al., 2003[53]; Bouillon et al., 2008[19]; Saponaro et al., 2020[139]).

Vitamin D is attached to its serum carrier, vitamin D binding protein (DBP) in the bloodstream. DBP is a highly polymorphic protein that has at least 120 different isoforms. Gc1f, Gc1s, and Gc2 are the three primary isoforms that have sparked the most interest. Their structural differences have an impact on DBP function, which is linked to a large number of clinical aspects. Studies confirm that polymorphism of specific DBP isoforms leads to a lower level of circulating DBP (Santos et al., 2019[138]). Serum calcium levels have been reported to be normal in patients with low levels of circulating DBP. The primary role of DBP is to keep a stable reservoir of circulating extracellular vitamin D metabolites in place. DBP is more effective in this role than albumin due to its stronger affinity for vitamin D metabolites, even though both DBP and albumin are filtered into urine and retrieved by megalin. 

Under normal physiological conditions, 1α,dihydroxyvitamin D binds to its nuclear receptor, the vitamin D receptor (VDR), and then to a retinoid X receptor (RXR), forming a VDR-RXR heterodimer that interacts with regulatory elements in the genome and regulates the transcription (Haussler et al., 1997[57]). VDR is a transcription factor that has been shown to influence the expression patterns of many genes (Khammissa et al., 2018[73]). The interaction of 1α,25-dihydroxyvitamin D with its intracellular receptors is also known to affect vitamin D-dependent gene transcription and activation of vitamin D-responsive elements and trigger various second messenger systems (Gil et al., 2018[48]). Vitamin D deficiency raises the risk and severity of several diseases, including obesity, insulin resistance, type 2 diabetes, hypertension, pregnancy issues, memory difficulties, osteoporosis, autoimmune diseases, malignancies, and systemic inflammatory illnesses (Garcia-Bailo et al., 2011[45]; Berridge, 2017[14]; Szymczak-Pajor et al., 2020[151]). 

The Endocrine Society recommends a daily dose of 400 IU of vitamin D for children aged 0 to one year and 600 IU for children aged one to eighteen years. The Society advises 1500-2000 IU for men and women over 18 years old, including nursing and pregnant women whose newborns are not getting enough vitamin D (Endocrine Society, 2017[41]). Vitamin D deficiency/insufficiency is a public health problem because it is independently connected with a greater risk of all-cause mortality. Vitamin D3 therapy with a daily dose of 500 U reduced the frequency of respiratory infections by two-thirds in patients with 25-hydroxyvitamin D levels less than 20 ng/ml, which typically leads to impaired absorption of vitamin D. Vitamin D sufficiency, a serum 25-hydroxyvitamin D level of at least 30 ng/mL reduced the risk for adverse clinical outcomes in patients with COVID-19 infection (Maghbooli et al., 2020[93]). Hypovitaminosis D was associated with a decline in muscular function and performance and increased disability (Berridge, 2017[15]; D'Amelio and Quacquarelli, 2020[31]). Vitamin D supplementation has been shown to boost muscle strength and speed in aged people (Halfon et al., 2015[55]; Berridge, 2017[15]). Vitamin D supplementation has been linked to a lower risk of falls due to direct effects on muscle cells (Ramasamy, 2020[128]; Wilson-Barnes et al., 2020[170]). On the other hand, excessive vitamin D levels might have harmful effects, such as kidney stones, renal impairment, malignancy, and possibly some indications of cardiovascular disease (CVD), especially when combined with a high calcium intake (Brouwer-Brolsma et al., 2013[23]).

**Figure 2:**
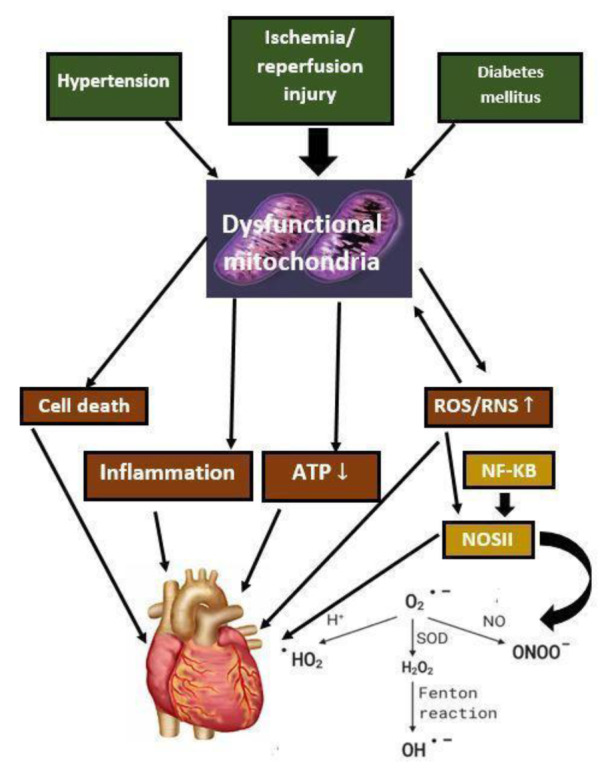
Vitamin D and potential linkage with critical biological functions, cardiac and mitochondrial diseases

The research focus on vitamin D has expanded beyond its recognized classic bone health benefits, including diabetes and cardiovascular, neurological, pulmonary, renal, and liver illnesses. Yet, several contradictory discoveries continue to emerge (Stokes and Lammert, 2016[149]). However, some controversies and uncertainties still exist in certain aspects related to a daily dose of vitamin D required in the general population to maintain normal levels of 25-hydroxyvitamin D, supplementation for metabolic bone diseases, ultraviolet-B induced cutaneous production of vitamin D, regulation of 25-hydroxyvitamin D metabolites in the liver, the definition of hypervitaminosis of D, hypovitaminosis in acute illness, requirements of vitamin D during reproduction, cellular and organ activities under vitamin D receptor influence, and possible links between vitamin D and major diseases (Minisola et al., 2019[108]; Giustina et al., 2020[50]). Keeping vitamin D levels in an optimal range allows it to improve several processes while avoiding the complications associated with overdose. The vitamin D fluctuation is widely associated with several diseases, including cardiovascular diseases (Ohsawa et al., 2000[115]; Brewer et al., 2011[22]), cancer (Hammad et al., 2013[56]; Weinstein et al., 2015[167]), immune system disorders (Jeffery et al., 2015[67]; Wang et al., 2017[163]), diabetes (Al-Timimi and Ali, 2013[5]), neuropsychiatric disorders (Kesby et al., 2011[72]) and several other diseases. The central focus of this review is to explore the potential of vitamin D, its association with mitochondrial dysfunction, oxidative stress, cellular damage, inflammation, and immune system (Jeffery et al., 2015[67]), calcium homeostasis linked with vitamin D, and cardiac diseases (Figure 2(Fig. 2)). 

## Role of Vitamin D in Mitochondrial Function/Dysfunction

Mitochondria are cell organelles with outer and inner membranes, though the inner membrane forms many folds known as cristae (Ashcroft et al., 2020[9]). The intermembrane space refers to the space between the outer and inner membranes, while the matrix refers to the space within the inner membrane (Ashcroft et al., 2020[9]). The mitochondria produce energy in the form of ATP. The reduction in ATP production is independent of changes in many parameters of mitochondrial machinery, including electron transport system (ETS) complexes I-V, citrate synthase, and cytochrome C oxidase (Ashcroft et al., 2020[9]). After the step-by-step transmission of electrons, the mitochondrial matrix actively pumps hydrogen ions into the intermembrane space. ATP synthase returns protons from intermembrane space to the mitochondrial matrix by passing them across an electrochemical gradient process. ATP is synthesized by coupling proton translocation with phosphorylation of ADP.

In addition to impacting muscle mass and functionality, evidence suggests that vitamin D in skeletal muscles may influence mitochondrial activity. In the case of vitamin D deficiency, mitochondrial respiration diminishes with a reduction in nuclear mRNA and protein (Kim et al., 2014[76]). When 1,25-dihydroxyvitamin D3 was administered to human primary myoblasts, mitochondrial activity improved, and the number of mRNAs encoding mitochondrial proteins increased by almost 80 percent (Ryan et al., 2016[137]). Vitamin D is needed to sustain the functioning of the mitochondrial respiratory chain (Consiglio et al., 2015[29]). The synthesis of the uncoupling protein (UCP), which regulates thermogenesis on the internal mitochondrial membrane, is similarly affected by vitamin D (Abbas, 2017[1]). In particular, the synthesis of ATP is reduced because of the decrease in vitamin D-dependent development of the electron transport chain complex I. Ashcroft et al., (2020[9]) found that VDR is required to maintain mitochondrial respiration at an optimal level in myoblasts and myotubes (Ashcroft et al., 2020[9]). The reduction in mitochondrial respiration in VDR-deleted myoblasts and myotubes as a result of reduced ATP. Further, vitamin D has no effect on mitochondrial ETC subunit I-V, citrate synthase, or cytochrome-c protein content in VDR-KD myoblasts and myotubes (Ashcroft et al., 2020[9]). The decrease in maximum oxidant ability without any alterations in ETS I-V protein expression was reported in an *in vivo* study with mice deprived of vitamin D (Habib et al., 2020[54]).

Most of the vitamin D research in humans focuses on protein synthesis and breakdown. There is growing evidence that vitamin D supplementation improves mitochondrial density and function (Sinha et al., 2013[146]; Rana et al., 2014[129]). Vitamin D supplementation enhances the balance between synthesis and breakdown of muscle protein, as well as mitochondrial density in an *in vivo *rat model study (Gogulothu et al., 2020[51]). To accomplish the immediate and intensive energy demands during exercise, the muscle stores phospho-creatinine (P-creatinine) (Bouillon and Verstuyf, 2013[20]). P-creatinine is the muscle's inorganic phosphate content. In vitamin D deficient patients, P-creatinine levels were decreased. However, it was restored after vitamin D treatment and exercise in randomized clinical trials (Wagner et al., 2013[161]). The slower rate of energy generation in the mitochondria of skeletal muscle could lead to a loss of muscle strength and a fast feeling of exhaustion during moderate activity (Latham et al., 2021[84]). P-creatinine is broken down during muscle contraction and generates creatine. The P-creatinine system generates a lot of ATPs, which is crucial when metabolic demand is high, such as during intense aerobic exercise, and other metabolic pathways cannot keep up with the need. In essence, P-creatinine fosters the short-term high level of energy needed during intense exercise. Ye et al. (2001[174]) observed a decrease in the P-creatinine /ATP ratio in a pig model of congestive heart failure (Ye et al., 2001[174]). Further, research shows that P-creatinine system damage occurs before contractile dysfunction, which reduces the energy reserve (Ingwall and Weiss, 2004[64]). Vitamin D could reverse P-creatinine fluctuation in contractile function by restoring mitochondrial function, and this could be a potential research target for further investigation. Furthermore, the activation of the vitamin D receptor protein has been confirmed to increase serum creatinine. Concern regarding this fact is that while serum creatinine levels are increased, the glomerular filtration rate decreases. However, a study found results to the contrary. The short-term activation of vitamin D receptors increases serum creatinine levels along with overall creatinine production and generates no detrimental effects on the glomerular filtration rate (Agarwal et al., 2011[2]). 

### The role of vitamin D receptors in mitochondrial function

The ubiquitous nature of VDR suggests the possibility of extensive impacts, prompting further research into the effects of vitamin D in several tissues in the human body (Omdahl et al., 2002[116]). In genomic results, VDR activation in the nucleus leads to cellular differentiation and proliferation (Sirajudeen et al., 2019[147]). Nongenomic effects leading to fast calcium influx within muscle cells could be attributed to a potential transmembrane receptor (Rebas et al., 2017[131]).

**Figure 3:**
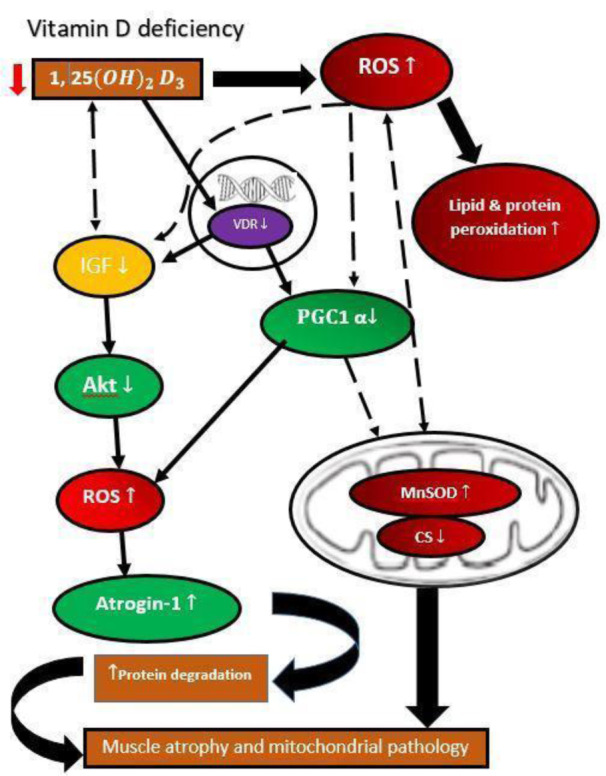
Effect of deficiency of vitamin D3 causing protein degradation and muscle atrophy

1,25-dihydroxyvitamin D controls the oxidative capacity by avoiding substantial changes in the density or amount of ETS protein. A recent study revealed that VDR knock-down (VDR-KD) in myotubes of C2C12 enhances the synthesis of mitochondrial fusion protein optic atrophy 1 (OPA1), which has been proposed as a compensatory strategy for restoring mitochondrial function (Ashcroft et al., 2020[9]). In mitochondria, OPA1 causes internal membrane fusion, resulting in improved mitochondrial oxidative capacity (Kushnareva et al., 2013[83]). The OPA1 protein expression was elevated with 1,25-dihydroxyvitamin D treatment in vitamin D deficient mice with statin-induced myopathy and human skeletal muscle cells (Ryan et al., 2016[137]; Ren et al., 2020[132]). Ricca et al. (2018[133]) studied the *in vitro* function of mitochondria by silencing VDR and reported the enhanced respiratory activity in silenced cells that was associated with increased reactive oxygen generation (ROS). The absence of the receptor eventually led to mitochondrial malfunction and cell death and slowed down cellular proliferation. These results indicate that VDR protects cells against excess breathing and ROS production that cause cell damage (as shown in Figure 3(Fig. 3)) (Ricca et al., 2018[133]).

Furthermore, a similar study by Ricciardi et al. (2015[134]) found that vitamin D and VDR signaling decreases mitochondrial respiration, serving as an important regulator for many essential bioenergetic metabolic pathways. The ability of vitamin D/VDR to regulate mitochondrial respiration allows the mitochondria to adapt to various metabolic states that arise during times of cell growth, signaling, and proliferation (Silvagno and Pescarmona, 2017[145]). To continue, the study conducted by Silvagno and Pescarmona (2017[145]) found that KO VDR mice have shown deficient calcium absorption leading to hypocalcemia, hypophosphatemia, secondary hyperparathyroidism, osteomalacia, and rickets. The same study also identified VDR as a mitochondrial energy expenditure regulator due to the finding that VDR KO mice have shown higher basal energy expenditure rates and increased levels of energy up-coupling in the form of the electron transport chain in mitochondria. Additionally, VDR KO mice were found to have a debilitating effect on the skin in regards to decreasing the efficiency of the barrier to a host of pathogens via disruption in the processes of lipid composition and secretion that help make the barrier effect.

The VDR protein was discovered with two anti-VDR antibodies, and the mitochondrial VDR disappeared by the VDR gene silencing in immortalized human keratinocytes (Consiglio et al., 2014[28]). VDR localization in mitochondria needs a specific mitochondrial import mechanism involving the import of cholesterol or the export of cytochrome C (Silvagno et al., 2013[144]). It's unclear how mitochondrial VDR affects gene expression, cation control, oxidative function, and tissue-specific activity in platelets or muscles. It also regulates several other nuclear receptors in mitochondria including estrogens, glucocorticoids, and thyroid hormone receptors (Psarra et al., 2006[124]). Mitochondria are generally acknowledged as having a function in forming reactive nitrogen, reactive oxygen species (ROS), and antioxidants. The antioxidants' defense mechanisms were shown to be enhanced in rickets (Doǧan et al., 2012[35]). Both metabolic and osteoporosis syndrome is associated with oxidative stress (Manolagas, 2010[95]). 

The lack of VDR leads to a decrease in cell proliferation, which is highly required during the cells that acquired higher VDR in the G0 and G1 phases of the cell cycle than in the M phase (Consiglio et al., 2014[28]). Consiglio et al. (2014[28]) found that cancer cells with silenced VDR have decreased the rates of proliferation, leading to the hypothesis that reduced VDR expression could be a possible cancer treatment. Silencing of VDR increased cytochrome C oxidase subunits II and IV transcript. VDR silencing inhibited the mevalonate pathway and histone acetylation levels, which are acetyl CoA-dependent biosynthetic pathways (Consiglio et al., 2014[28]). Inhibiting histone acetylation decreases gene transcription levels, and since acetyl CoA is a key molecule in metabolism, various pathways have the potential to be affected by VDR. These findings suggest that VDR regulates the mitochondrial respiratory chain activity, the acetyl-CoA pathway, the TCA cycle, and biosynthetic pathways of cell development.

Researchers investigated the significance of vitamin D in maintaining the *in vivo* activity of mitochondria by utilizing a known diet-induced vitamin D deprivation model in mice C57BL/6J. The study with a diet-induced vitamin D deficiency model of mice(C57BL/6J) results in reduced mitochondrial respiration in skeletal muscle that could lead to muscle fatigue and performance deficits (Ashcroft et al., 2021[10]). Vitamin D (calcitriol) increases intramyocellular lipid (IMCL) accumulation and oxygen consumption rate, which is driven by mitochondrial complex II in C2C12 myotubes, and this increase is at least partially mediated by a protein, Perilipin 2 (PLIN2) (Schnell et al., 2019[140]). Kolleritsch et al. (2020[80]) reported that the low cardiac perilipin is linked to reduced mitochondrial fission and could be used to inhibit the emergence of lipotoxic cardiomyopathy (Kolleritsch et al., 2020[80]). An increase in myocardial lipid storage and decreased cardiac performance were observed after myocardial infarction in people with PLIN2 deficiency(Mardani et al., 2019[98]).

## Vitamin D, Mitochondria, and Cardiac Diseases

Cardiovascular diseases, including heart failure, aortic aneurysmal heart disease, peripheral artery disease, hypertension and atherosclerosis, coronary artery disease, myocardial infarction, hypertrophy, cardiomyopathy, and cardiac fibrosis, are significant causes of morbidity and mortality (Drazner, 2011[37]; Rai and Agrawal, 2017[126]; Elgendy et al., 2019[40]). These illnesses are linked to low levels of vitamin D, and supplementing with vitamin D is an effective treatment option (Wang et al., 2008[164]). Myocardial infarction is the prominent cause of morbidity and mortality in the world. Lee et al., reported that vitamin D3 inhibits oxidative stress and regulates mitochondrial activity to reduce hypoxia/reoxygenation (H/R)-induced apoptosis in a mouse model (Lee et al., 2020[86]). They also stated that vitamin D3 exerts cardioprotective effects and reverted H/R-induced mitochondrial fission and mitophagy by inhibiting mitochondrial fission proteins, phosphorylated dynein-related protein 1 (pDrp1), and mitochondrial fission factor (Mff) (Lee et al., 2020[86]). Mitochondrial fusion and fission are dynamic events, which play a significant role in mitochondrial and cellular quality control processes (Youle and Van Der Bliek, 2012[175]). In mitochondrial fusion, two small healthy mitochondria fuse and form a mitochondrion that can produce ATP for cellular function. In another way, the damage to mitochondria by fission and mitophagy will be eliminated (Westermann 2010[169]; Youle and Van Der Bliek 2012[175]; Chidipi et al., 2021[26]). Vitamin D receptors are expressed in the cardiovascular system and activated by modulating the renin-angiotensin system, inflammation, and fibrosis against myocardial hypertrophy and hypertension (Gardner et al., 2013[46]). The renin-angiotensin-aldosterone system is especially susceptible to vitamin D since it negatively regulates renin and is correlated with a decrease in blood pressure and left ventricular hypertrophy. In a study by Carrara et al., patients with hypertension and vitamin D deficiency were given a weekly dose of cholecalciferol for two months. At the end of the study, all participants have decreased levels of plasma renin and aldosterone (Carrara et al., 2014[25]). These findings can potentially improve outcomes for patients with hypertension. Furthermore, a novel study by Tomaschitz et al., confirmed this by concluding that lower vitamin D levels correlate to an upregulation of the renin-angiotensin-aldosterone system, inevitably leading to hypertension (Tomaschitz et al., 2010[155]). This provides evidence for vitamin D has a strong effect on both the cardiovascular and renal systems. To continue, Diez et al. tested a daily dose of 30 ng/kg of vitamin D in a modified VDR ischemia-reperfusion (I/R) rats model. They also found reversed ischemia-reperfusion changes by restoring myocardial vitamin D receptor levels and prolonging action potentials (Diez et al., 2015[33]). Lack of VDR causes increased left ventricle (LV) mass and elevated levels of atrial natriuretic peptide coupled with an imbalance of homeostasis, and metalloproteases of heart and fibroblasts. These findings suggest that vitamin D deficiency may be associated with vascular dysfunction, arterial steadiness, and enlargement of the LV. Sufficient or insufficient vitamin D levels may have a role in the development of cardiovascular disease (Khan et al., 2016[74]). VITAL (VITamin D and OmegA-3 TriaL) and ViDA (Vitamin D Assessment) are two extensive, randomized control studies conducted to study the effects of vitamin D supplementation on CVD outcomes (Manson et al., 2012[96]; Scragg, 2020[142]). The VITAL is a randomized clinical trial with 25,871 US subjects who found that daily dietary supplementation of vitamin D3 (2000 IU) or omega-3 fatty acids (1 gram) reduces the risk of cancer development, heart disease, and stroke in people without previous history of these illnesses (Manson et al., 2012[96]). ViDA study found that vitamin D supplementation does not affect the primary outcomes such as cardiovascular disease, acute respiratory infections, non-vertebral fractures, falls, and all cancers (Scragg, 2020[142]).

### Effect of vitamin D on oxidative stress

**Figure 4:**
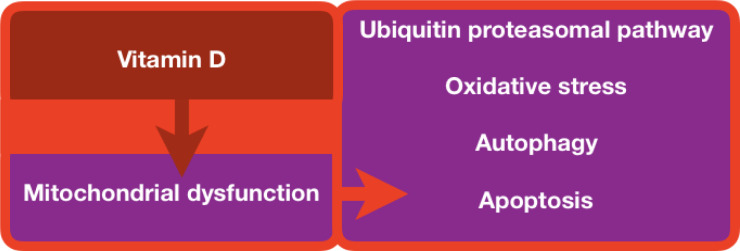
Vitamin D links mitochondrial dysfunction and the consequences on major biological processes and functions.

Increased ROS levels cause oxidative stress. During myocardial ischemia/reperfusion (I/R), mitochondria generate ROS as a result of aerobic metabolism (Chouchani et al., 2014[27]). Vitamin D3 can boost endothelial cell proliferation and suppress apoptosis via increasing endothelial nitric oxide synthase (eNOS) expression and nitric oxide (NO) production (Molinari et al., 2013[110]). The phosphorylation of NOS in the heart is a crucial adjunct for myocardial perfusion following ischemia and myocardial contractility, oxygen consumption, hypertrophic remodeling, apoptosis, and myocardial regeneration in cardiac cells (Ahmad et al., 2018[3]; Farah et al., 2018[43]). NOS also improves the anticoagulant and anti-thrombogenic capacity of vascular endothelium, maintains vascular tone, and prevents the proliferation of vascular cells (Rajendran et al., 2013[127]). Nitric oxide controls various signaling molecules such as soluble guanylate (sGC), cytochrome C oxidase, and hemoglobin by binding it to iron heme in the metalloproteins (Tsai et al., 2012[156]). The interaction of NO with heme of sGC in smooth muscle cells near the endothelium catalyzes guanosine triphosphate (GTP) into guanosine monophosphate (cGMP), which is the primary mechanism for the NO action (Tsai and Kass, 2009[157]). Smooth muscle membrane hyperpolarization is regulated by cGMP-dependent protein kinases, which limit cytosolic calcium flow by enhancing calcium-dependent potassium opening and cell hyperpolarization (Koh et al., 1996[79]). Hu et al. investigated the mechanisms involved in the inhibition of myocarditis by vitamin D in experimental autoimmune myocarditis (EAM) mice model (Hu et al., 2016[62]). The treatment of vitamin D reverted left ventricular dysfunction (ejection fraction and fractional shortening), apoptosis, and autophagy (Hu et al., 2016[62]). It is also known that vitamin D may improve heart function by suppressing inflammatory cardiac infiltrations, lowering apoptosis of cardiomyocytes, and modulating autophagy (Hu et al., 2016[62]). Even though research has linked ROS to heart tissue damage, there is no cure at present (Figure 4(Fig. 4)). Further studies may yield new therapeutic insights for I/R (Granger and Kvietys, 2015[52]). Furthermore, vitamin D has the potential to combat oxidative stress not only in cardiac myocytes but also in photoreceptors of the eye. In a study conducted by Tohari et al., mouse cone cell lines were subjected to oxidative stress and then given a vitamin D treatment (Tohari et al., 2016[154]). The results showed that the oxidative stress in the cone cells was reversed and this finding has the potential to impact human patients with photoreceptor diseases. 

### Vitamin D and inflammation 

Vitamin D metabolites affect immune and inflammatory cell differentiation and production of cytokines, which means vitamin D metabolites play an essential role in the development of atherosclerosis and other vascular inflammation-related disorders (Martens et al., 2020[99]). 25-hydroxyvitamin D and its active hormonal form, 1,25-dihydroxyvitamin D, are required for human physiological processes, including reducing inflammation and intracellular oxidative stress. Vitamin D is a crucial regulator of systemic inflammation, oxidative stress, and mitochondrial respiratory function in humans, as well as the aging process (Bhatti et al., 2017[17]). Vitamin D deficiency and atherosclerosis are both common diseases and there is reason to believe that a correlation exists between the two. Atherosclerosis is characterized as a lipid storage disease known as vascular wall inflammation (Kim et al., 2008[77]; Marchio et al., 2019[97]; Wimalawansa, 2019[171]). The lipids have been deposited, and T-cells and macrophages are accumulated due to the endothelium reaction (Mosser and Edwards, 2008[112]; Gibson et al., 2018[47]). Reactive oxygen species are of central importance, which may lead to the oxidation of lipids, including low-density lipoproteins and polyunsaturated fatty acids, which are deposited in the vascular wall, and harm cellular components directly (Leopold and Loscalzo, 2008[88]; Rafieian-Kopaei et al., 2014[125]). Nitric oxide, mentioned previously, serves a protective function in the endothelium. Interestingly, vitamin D increases endothelial nitric oxide thereby helping to maintain the vasculature and avoid atherosclerosis (Menezes et al., 2014[101]). Vitamin D deficiency is common globally and seems to be implicated in several stages in the pathophysiology of atherosclerosis (Kassi et al., 2013[71]; Latic and Erben, 2020[85]). Atherosclerotic lesions are generated that may rupture and lead to vascular lumen blockage via the activity of multiple cytokines (Lusis, 2000[91]; Badimon et al., 2012[11]). Vitamin D suppresses the absorption of cholesterol by macrophages, and in the case of vitamin D deficiency, macrophagic cholesterol uptake occurs and is finally deposited into endothelial spaces, which promotes atherosclerosis (Pludowski et al., 2013[122]; Cyprian et al., 2019[30]). Diminished high-density lipoproteins and apolipoprotein A-1 ratios, that cause atherosclerosis, were combined with a deficiency in vitamin D (Weng et al., 2013[168]). Vitamin D effectively reduces the intracellular NF-κB levels to reduce atherosclerosis development (Legarth et al., 2019[87]). Mitochondrial dysfunctions were detected in atherosclerosis, including downregulation of mitophagy similar to autophagy, which can eliminate the damaged part of the mitochondria (Yang et al., 2020[173]; Poznyak et al., 2021[123]) (Figure 4(Fig. 4)). However, it is unclear whether vitamin D regulates mitochondrial dysfunction in atherosclerosis development (Mandarino et al., 2015[94]; Poznyak et al., 2021[123]). Moreover, VDR being found on various tissue types, including brain and pancreas tissue, links vitamin D and cardiovascular diseases such as atherosclerosis and hypertension stronger (Menezes et al., 2014[101]). In addition to the direct relationship between vitamin D deficiency and heart disease, the deficiency can indirectly exacerbate cardiac symptoms by interfering with endocrine processes that impact cardiac function. One example of this indirect link is the ability of vitamin D to decrease insulin resistance, which ultimately benefits patients with atherosclerosis and hypertension by decreasing the activity of lipoprotein lipase, leading to a decrease in the level of lipoproteins and LDLs in the blood (Menezes et al., 2014[101]).

### Vitamin D and calcium homeostasis

Vitamin D deficiency has been associated with an increased mortality rate, particularly cardiovascular mortality (Heath et al., 2019[58]). The calciotropic hormones, including vitamin D, parathyroid hormone, and calcitonin, are responsible for maintaining calcium homeostasis within normal ranges (Mundy and Guise, 1999[113]). Vitamin D regulates intracellular calcium and ROS (Duchen, 2000[38]). The fluctuation of intracellular calcium regulates mitochondrial calcium and health (Bagur and Hajnóczky, 2017[12]). Cardiac cell contractual characteristics are primarily regulated by the direct contact with the calcium, known as the calcium-induced calcium release mechanism (Eisner et al., 2017[39]). The cardiac contractile proteins, actin, and myosin are regulated by the intracellular levels of calcium (Rüegg, 1998[136]; Kuo and Ehrlich, 2015[82]). The extracellular homeostasis of calcium influenced by vitamin D alters intracellular calcium and may impact heart cell contractility indirectly (Weber et al., 2008[165]). Pfeifer et al. studied older women with vitamin D deficiency who were supplemented with calcium and 20 μg of vitamin D3 as a daily dose and found an increase in serum 25-hydroxyvitamin D of 20 nmol/l, a 9.3 % de-crease in systolic blood pressure, and a 5.4 % decrease in heart rate compared with those supplemented with calcium alone (Pfeifer et al., 2001[120]). They also concluded that vitamin D3 and calcium intake could contribute to the pathogenesis and progression of hypertension and cardiovascular disease in older women (Pfeifer et al., 2001[120]). The alteration of intracellular calcium could affect the mitochondrial function via VDR. Vitamin D regulates mitochondrial calcium homeostasis. Mitochondrial calcium has additional vital functions, such as mitochondrial metabolic control, ATP generation, and cell death (Giorgi et al., 2012[49]). Another clinical study with 3258 participants showed that severe and moderate vitamin D deficiency (19.0 and 33.3 nmol/l) leads to a higher rate of cardiovascular death compared to the patients with normal levels (71.0 nmol/l) over 7 years (Murr et al., 2012[114]).

The endocrine hormone 1,25-dihydroxyvitamin D is produced in response to dietary calcium intake and physiologic states such as growth, aging, and menopause (Fleet, 2017[44]). Most of the molecular activities of 1,25-dihydroxyvitamin D on calcium-regulating target tissues are mediated via transcription regulated by the vitamin D receptor (Pike and Christakos, 2017[121]). Calcium homeostasis may be controlled by blood calcium levels of the necessary ranges, vitamin D endocrine regulates the total calcium homeostasis of the body, and the regular dietary calcium intake helps regulate the metabolism of vitamin D (Fleet, 2017[44]). The primary role of vitamin D is in regulating intestinal calcium absorption, urinary calcium excretion, and bone metabolism. To achieve this goal, these regulatory events occur in coordination with numerous tissues, including the intestine, kidney, bone, and parathyroid gland (Fleet, 2017[44]; Bhattarai et al., 2020[16]).

## Vitamin D and Reproductive Health

**Figure 5:**
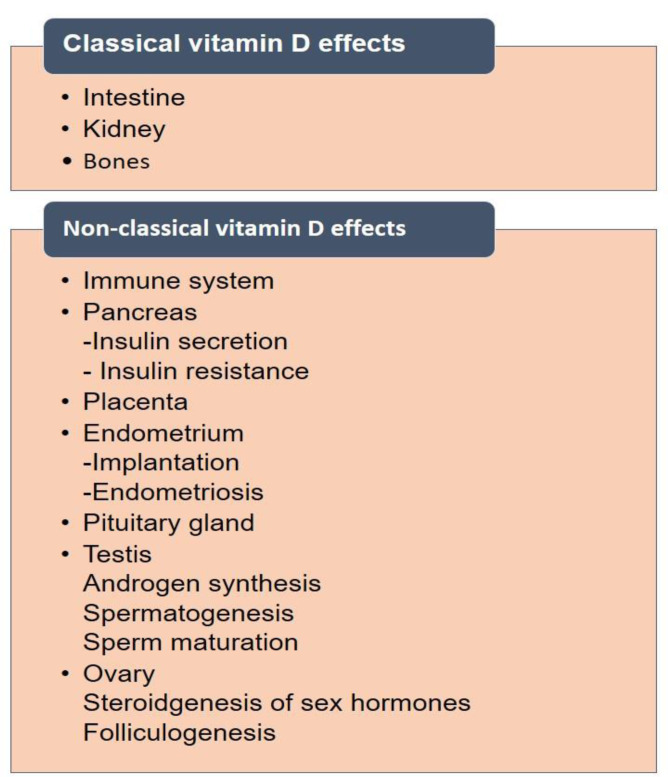
Classical and non-classical effects of vitamin D on human health

The link between vitamin D and human reproduction is precious. There are two types of effects covered under vitamin D levels and their effects on human organs. These are classical and non-classical effects (Figure 5(Fig. 5)). There are several reports available on the proven vital link between vitamin D levels and reproductive health in humans.

### Role in the female reproductive system

The pivotal role of vitamin D is well studied in the female reproductive system. There are three important phases of reproductive women's life span. These are menarche, adolescence, reproductive period, and menopause. Poor vitamin D status in the developing stage is a serious matter of concern as it is critical for optimal bone mineral status in the developing skeleton. Literature suggested that the Dietary Reference Intake (DRI) is very poorly distributed among growing girls. It was found that a total of 50 % of girls aged between 9-13 years and 32 % of girls aged between 14-18 years are only meeting the recommendation for vitamin D (200 IU/d or 5 mg/d) (Moore et al., 2004[111]). In adolescents, this deficiency leads to decreased absorption of dietary calcium which results in an altered form of the growth and poor mineralization of the skeleton. Sometimes this deficiency leads to the generation of secondary hyperparathyroidism, and a higher risk of developing bone abnormalities (Holick, 2004[60]). Vitamin D plays a critical and potential role in the modulation of obesity, energy metabolism, and insulin secretion in adolescent stage of females (Skinner et al., 2003[148]). Another aspect of vitamin D deficiency is linked with puberty which is a time of dramatic developmental changes in the body in a sequential manner to reach mature adult reproductive stages. It is well known that the timing of menarche generally depends on temperature, sun exposure, and socioeconomic status in society, but in some ways it is directly related to a geographic gradient of specific sun exposure habits, ultimately leading to vitamin D status at this stage. Hence, we can conclude that vitamin D status is linked with menarche. Finally, vitamin D status could indirectly affect the timing of menarche through its effect on obesity in developing girls. Some physiological and biochemical pathways might play an important role in secretion of adipose-derived hormones, but it is unclear whether these hormones derived from adipose tissues could alter in response to vitamin D supplementation or not (Yura et al., 2000[176]; Maetani et al., 2009[92]; Donoso et al., 2010[36]). It was reported that Insulin-like growth factor-1 (IGF-I) may regulate the releasing of sex hormones. Vitamin D receptors have been shown in different parts of the brain including the hypothalamus thus it may be positively related to the age at menarche, and its insufficiency was associated with earlier menarche through neuroendocrine regulation of the gonadotropic axis (Zhen et al., 1997[177]; Eyles et al., 2005[42]; DiVall and Radovick, 2008[34]; Breen et al., 2011[21]; Villamor et al., 2011[159]).

It was reported that vitamin D regulates the expression of a large number of genes involved in the reproductive tissues of the female reproductive system. Several tissues of the endocrine and reproductive system are having VDR. In females, vitamin D is critically involved in the physiological functions of ovarian follicles. It was studied that human ovaries contain granulosa cells. The nuclei and cytoplasm of these cells are abundantly containing vitamin D receptors. This indicates that vitamin D plays an important role in the female reproductive system (Thill et al., 2009[153]).Thus, we can say that vitamin D deficiencies directly or indirectly play role in issues of subfertility, endometriosis, polycysticovary syndrome (PCOS), preeclampsia, preterm delivery, gestational diabetes, and bacterial vaginosis. Hence, optimal vitamin D levels in the reproductive phase and throughout a woman's life are always important. It was also reported that vitamin D induces somehow the secretion of important hormones progesterone, estrone, and estradiol secretion in ovarian cells either independently or synergistically with insulin.

In the case of follicular development studies, it has been concluded that vitamin D might promote the differentiation and development of human granulosa cells, thus playing an important role in human follicular development (Merhi et al., 2008[105], 2012[106], 2014[103]; Merhi, 2009[104]; Irani and Merhi, 2014[65]).

Most of the sex hormones are derived from cholesterol which works as the common precursor and can be obtained either through dietary supplements or *de novo* synthesized from acetyl CoA. The production process of these hormones is controlled by multiple enzymes. It was evident from published reports that the expression and activity of some of these enzymes were affected by vitamin D (Merhi et al., 2014[103]). It was also found that in human ovarian cells, the production of vital hormones like progesterone, estrogen, estrone, and insulin-like growth factor-binding protein 1 has increased under the direct influence of vitamin D levels. It was also reported that 1,25-dihydroxyvitaminD3 strongly stimulated the production of the hormones estrogen and progesterone in the human placenta (Barrera et al., 2007[13]).

It was reportedin the case of female rats that a low level of vitamin D leads to a 75 % decrease infertilitywhich further leads to complications in pregnancy. Sometimes, this deficiency may link with uterine hypoplasia and impaired folliculogenesis. Calcium homeostasis is maintained by vitamin D sufficient level in the reproductive phase which finally modulates the estrogen biosynthesis (Panda et al., 2001[118]; Sun et al., 2010[150]; Wojtusik and Johnson, 2012[172]). Calcium repaired fertility was studied in the case of animals which was achieved by vitamin D and a diet supplemented (Johnson and DeLuca, 2001[69]; Anagnostis et al., 2013[7]).

Pregnancy and lactation are two very precious stages for every female. It was reported that the active form of vitamin D was highly required to increase the intestinalabsorption of calcium and the mobilization of maternal bones. A total of approximately 30 g of calcium is absorbed by human embryos. Skeleton contains 99 % of this calcium. Almost 150 mg/kg/day of calcium is transferred by placenta during the last trimester of pregnancy (Kovacs, 2008[81]). Vitamin D deficiency is prevalent among pregnant women. It was reported during pregnancy stages, increase in the plasma vitamin D levels could contribute to the reductionin plasma calcium level and may result from increased metabolism of mothers or increased utilization of vitamin D by the fetus (Lerchbaum and Obermayer-Pietsch, 2012[89]).

It was studied that maternal vitamin D levels and the prevalence of bacterial vaginosis among pregnant women are directly linked with each other. Bacterial vaginosis has been reported to disrupt the normal balance of vaginal flora, leading to increased growth of anaerobic bacteria responsible for the secretion of inflammatory cytokines, prostaglandins, and phospho-lipase A2 (Allsworth and Peipert, 2007[4]).

Calcium status plays a very critical role in the initiation of labor and also plays a role in smooth muscle function in early labor. The level of serum calcium is generally regulated by vitamin D levels (Papandreou et al., 2004[119]). Pregnant women with low levels (<37.5 nmol/l) of 25(OH)D3 delivered more than 4 times by cesarean section compared to women with 37.5 nmol/l or greater with normal delivery (Merewood et al., 2009[102]). Vitamin D is essential for the maintenance of calcium homeostasis and a role in the initiation of early labor. Vaginal delivery was severely affected due to the poor maternal vitamin D status which might reduce the strength of the pelvic musculature in pregnant women (Scholl et al., 2012[141]). Fetal development and programming in pregnant women is directly linked with 3000 genes that are stimulated by vitamin D levels (Kho et al., 2010[79]). Mother and child health is linked with normal vitamin D levels. The deficiency during pregnancy of vitamin D leads to chronic diseases in later stages of child. Most of them are like wheezing and asthma, schizophrenia, multiple sclerosis, type 1 diabetes mellitus, and insulin resistance (Altschuler, 2001[6]; Hyppönen et al., 2001[63]; Camargo et al., 2007[24]; Devereux et al., 2007[32]; Zipitis and Akobeng 2008[178]; Mirzaei et al., 2011[109]).

### Vitamin D levels and their role in infertility

Approximately 15 % of the couples are severly affected by infertility disorders due to poor level of vitamin D. These are due to the various problems like polycystic, ovary syndrome, endometriosis infertility, myoma infertility, male infertility, premature ovary failure. These problems can be cured by maintaining the normal levels of vitamin D. The literature demonstrates that low vitamin D levels very often lead to PCOS compared to women with normal levels (Li et al., 2011[90]; Wehr et al., 2011[166]). The deficiency is also linked with insulin resistance, obesity, and metabolic syndromes. These are commonly observed in PCOS which leads to ovulatory dysfunction (Hosseinpanah et al., 2014[61]). Menstrual irregularity can be removed by proper supplementation of vitamin D. It might be critically involved in improvement of follicular development, and pregnancy rate in women with PCOS (Rashidi et al., 2009[130]; Ott et al., 2012[117]).

### Role in male reproductive system

Role of calcium is essential in the male reproductive system. This is highly required and crucial for spermatogenesis, and sperm motility. It was found that there is a direct role of vitamin D in semen quality and spermatogenesis which works as a modulator of calcium metabolism.

The basis of the interplay between vitamin D and reproduction lays on the presence of both.

**Figure 6:**
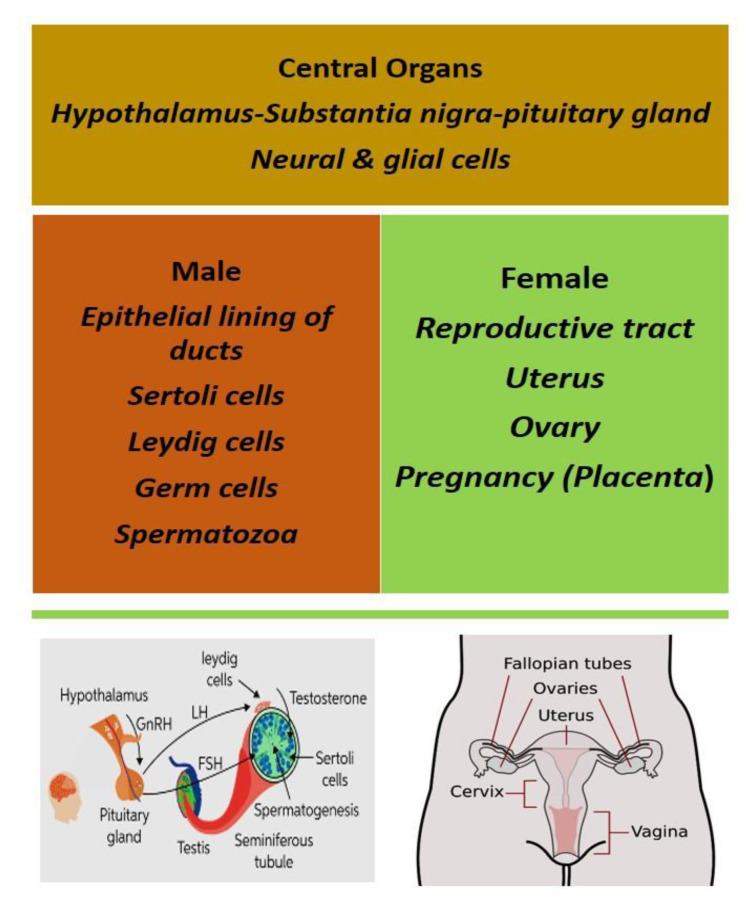
Vitamin D receptor (VDR) in both central and peripheral reproductive organs of both males and females

In the rat, vitamin D receptors (VDR) and 1α-hydroxylase (CYP27B1) have been reported to play important roles in various tissues of both sexes, but particularly in the rat testis (Hirai et al., 2009[59]). In case of human, it was reported that VDR are found in testis, epididymis, prostate, seminal vesicles, and Leydig cells. Although when it was compared with others, the expression level was found different, which was slightly higher in epididymis and seminal vesicles (Blomberg Jensen et al., 2010[18]). Cholesterol efflux in human sperm was regulated by vitamin D molecules which enhanced the sperm bioavailability. It was studied that cytoplasm of epithelial cells of the epididymis and ductal prostate epithelium was encountered with vitamin D receptors (Walters 1984[162]). The key role of few enzymes is well established in the function of vitamin D in various tissues. These enzymes are located either in the endoplasmic reticulum (ER) (e.g., CYP2R1) or in the mitochondria (e.g., CYP27A1, CYP27B1, and CYP24A1) (Figure 6(Fig. 6)).

Meanwhile, it was reported that CYP2R1 and CYPB1 play a very important role in all tissues of the reproductive tract. CYPR1 gene expression might play some significant role in reducing testicular damage (Menegaz et al., 2009[100]). It was supported by literature that the number and motility of sperm was directly linked with the protective effect of vitamin D from oxidative stress and cellular toxicity (Kägi et al., 1988[70]). Vitamin D plays a crucial role in the process of spermatogenesis and steroidogenesis through the induced expression of calcium-binding protein CaBP28k in the testis (Shahbazi et al., 2011[143]). Recently, it was reported that severe hypo-spermatogenesis or idiopathic sertoli cell-only syndrome (SCOS) in males was directly linked with lower plasma 25(OH)D concentrations despite the normal levels of total testosterone and estradiol (Aquila et al., 2009[8]; Rittenberg et al., 2011[135]). Thus, it was concluded by researchers that sperm motility and progressive motilityis correlated with serum levels of 25(OH)D. Vitamin D deficiency (<10 ng/ml) in males results in a lower proportion of motile, progressive motile, and morphologicallynormal spermatozoa (Jensen et al., 2011[68]). To evaluate the positive role of vitamin D supplementation in men's infertility, further advanced investigations are highly anticipated.

## Conclusion

There is significant evidence that lack of vitamin D contributes to the development of heart failure (Zittermann et al., 2006[179]). Vitamin D promotes mitochondrial homeostasis and prevents protein oxidation, lipid peroxidation, and DNA damage caused by oxidative stress. Autophagy, mitochondrial malfunction, inflammation, oxidative stress, epigenetic modifications, DNA abnormalities, and calcium and ROS signaling changes are all known to be regulated by vitamin D. The excess vitamin D may lead to calcification. Therefore, proper dosages of vitamin D are required to treat patients in clinics. In several patients, low blood levels of 25-hydroxyvitamin D lead to increased mortality, especially sudden cardiac death and coronary illness. In this review, we emphasized the potential role of vitamin D in critical biological processes and the functions of explorations of the biological components in mitochondrial-associated cardiac diseases.

In summary, vitamin D supplementation in humans plays a significant role in supporting mitochondrial health and regulating cardiac disease progressions. Detailed mechanical inquiries are necessary to light the manifestation of vitamin D in mitochondrial function and cardiac health.

## Notes

Shabana Bibi and Viajaya Nirmala Pangi (School of Life and Health Sciences, Adikavi Nannaya University, Rajahamahendravaram, Andhra Pradesh, India; E-mail: vijaya.nirmala3@gmail.com) contributed equally as corresponding author.

## Declaration

### Author contributions

Conceptualization, writing - original draft preparation was performed by A.M.R.; M.I.; S.U.; S.J.; and S.K.G.; editing and revision of original draft have been performed by H.C.; S.B.; I.S.; V.N.P; and M.M.A-D. All authors have read and agreed to the published version of the manuscript.

### Conflict of interest

The authors declare no conflict of interest.

### Funding

No funds, grants, or other support were received for this paper.
